# Switch to raltegravir-based regimens and HIV DNA decrease in patients with suppressed HIV RNA

**DOI:** 10.7448/IAS.17.4.19791

**Published:** 2014-11-02

**Authors:** Claudia Bianco, Genny Meini, Barbara Rossetti, Silvia Lamonica, Annalisa Mondi, Simone Belmonti, Luri Fanti, Nicoletta Ciccarelli, Simona Di Giambenedetto, Maurizio Zazzi, Andrea De Luca

**Affiliations:** 1 Infectious Diseases Unit, Azienda Ospedaliera Universitaria Senese, Siena, Italy; 2 Medical Biotechnology Department, University of Siena, Siena, Italy; 3 Clinic of Infectious Diseases, Catholic University of Sacred Heart, Roma, Italy

## Abstract

**Introduction:**

Raltegravir intensification is associated with an increase in 2-LTR episomal HIV DNA= circles, indicating a persistent low-level replication, in some individuals in ART with suppressed HIV RNA. We aimed at monitoring residual plasma HIV RNA and cellular HIV DNA in virologically suppressed patients switching to a raltegravir-based regimen.

**Materials and Methods:**

Forty-six HIV-infected subjects on PI or NNRTI based-regimens, with plasma HIV RNA level <40 copies/mL for ≥6 months and CD4 >200 cells/µL for ≥12 months were enrolled. Thirty-four patients switched to raltegravir-based regimen (RASTA study group) and 12 continued a PI or NNRTI based-regimen (control group). Ultrasensitive HIV residual viremia and total PBMC HIV DNA were assessed at baseline (W0), 24 (W24) and 48 (W48) weeks. HIV RNA levels were determined by an ultrasensitive test derived from a commercial real time PCR (limit of detection 5 copies/ml). A real time PCR was used to quantify HIV DNA copy numbers in PBMCs.

**Results:**

At W0, HIV DNA was detected in all patients while at W48 it was detectable in 82.3% of RASTA group vs 100% of controls (p=0.01). The difference between the average values of HIV DNA log_10_ copies/10°6 CD4 at W0 (median 3.11, IQR 2.70–3.45) and W48 (median 2.87, IQR 2.24–3.38) was statistically significant for RASTA group (p=0.035). Male gender (mean difference −0.37 log_10_ copies/10°6 PBMC, p=0.023) and previous PI based-ART (mean difference +0.39 log_10_ copies/10°6 PBMC, p=0.036) were predictive of HIV DNA level at W0. After adjusting for previous PI based-ART, male gender was the only variable independently associated with HIV DNA size at W0 (mean difference −0.326 log_10_ copies/10°6 PBMC, 95% CI −0.641, −0.011 p=0.043). Ultrasensitive HIV-1 RNA was detectable at W0 in 50% of RASTA group versus 66.7% of controls and at W48 in 32.4% versus 45.5%, respectively. No differences were found between HIV RNA levels at W0 and W48 within and between the two groups.

**Conclusions:**

Switching to raltegravir-based regimens may be associated with a decrease of HIV reservoir, as measured by total PBMC HIV DNA. A larger sample size is required to confirm this finding.

